# Commemorating 85 years of publications on Cannabis by Archives of Toxicology

**DOI:** 10.1007/s00204-021-03079-z

**Published:** 2021-05-30

**Authors:** Hermann M. Bolt, Jan G. Hengstler

**Affiliations:** Leibniz Research Centre for Working Environment and Human Factors (IfADo), Ardeystr. 67, 44139 Dortmund, Germany

Eighty-five years ago, the first report on a human *Cannabis indic*a intoxication appeared in this journal (Baker-Bates and Epple 1936). At that time, *Archives of Toxicology* was named “*Fühner-Wielands Sammlung von Vergiftungsfällen*” and was specifically devoted to the documentation of human intoxications by both natural and man-made agents.

The case of intoxication that was reported had occurred in 1933 in Liverpool, having received previous notes in the Liverpool Echo (Fleming 1935), the Police Journal (Anonymous 1934) and the Lancet (Baker-Bates 1935); the circumstances of the case had made a popular appeal (Fleming 1935). The historical and very unusual report shall be quoted verbatim: “A young man having read about Indian hemp in the Chemistry of Common Life by JFW Johnston (1855), which describes it as “increaser of pleasure, the exciter of desire, the cementer of friendship, the laughter-mover and the causer of reeling gait”, separated the hemp seeds from the parrot food and planted them in his garden during June. In September, when the plants were about four or five feet high and flowering, he plucked the leaves and tops, dried and chopped them and made them into cigarettes; these he smoked on several occasions and experienced mild symptoms of cannabis intoxication—e.g., loss of sense of time and space, vivid dreams or hallucinations and subsequent drowsiness. Incredulous about his experiences, his fiancée, aged 22, smoked—and to some extent inhaled—about two-thirds of a cigarette, made from the top of a fruiting plant (Baker-Bates 1935; Baker-Bates and Epple 1936).” The subjective and objective medical symptoms experienced by this young woman were described in detail. Finally, “The hot summer of 1933 may have been the reason why the seeds grew so well in England. There is a possibility that if the fact that hemp seeds could be grown with ease in England were widely known, hemp-smoking might become a national menace” (Baker-Bates 1935).

When this prophecy had become true, the abuse of marihuana triggered scientific activities on chemical analysis and biomonitoring (Seifert and Geldmacher 1955; Machata 1969; Hackel 1972; Reiß 1972). Later, synthetic cannabinoids were consumed as a surrogate of marihuana owing to their non-detectability with commonly used biomonitoring testing methods and to their strong cannabimimetic effects. Various toxic effects of such compounds were described (Koller et al 2013; Bileck et al 2016; Russo et al 2019; Tomiyama and Funada 2021).

The first synthetic cannabinoids were developed in the second half of the twentieth century to study human endocannabinoid receptor systems. However, today, synthetic cannabinoids represent the largest and most structurally diverse class of designer drugs, and some of these compounds are similar to phyto- and endocannabinoids. Synthetic cannabinoids are often referred to as ‘‘*Spice*,’’ based on the first branded synthetic cannabinoid product. They are commonly applied to dried herbs that mimic cannabis (Luethi and Liechti 2020).

Meyer (2016) pointed out that synthetic cannabinoids with a high affinity and intrinsic activity at cannabinoid CB1 receptors exert stronger physiological and psychological effects than tetrahydrocannabinol (THC), which may be in line with their high potential to trigger psychotic-like symptoms. From a clinical point of view, intake of synthetic cannabinoids should, therefore, be considered in patients known to abuse drugs and presenting psychiatric symptoms.

The endocannabinoid system is involved in various physiological functions, including cognition, behaviour, memory, motor control, pain sensation, appetite, cardiovascular parameters, gastrointestinal motility, and immunoregulation. The term ‘‘cannabinoid’’ refers to a class of compounds that are produced by *Cannabis sativa* and *Cannabis indica*, and endogenous and exogenous ligands that interact with G protein-coupled cannabinoid type receptors (CB1 and CB2). CB1 receptors are mainly expressed in the brain and modulate neurotransmitter signaling, whereas CB2 receptors are abundant in immune tissues (Luethi and Liechti 2020).

Luethi and Liechti (2020) also presented a compilation of reported clinical symptoms. The most common adverse effects of synthetic cannabinoids include agitation, drowsiness, dizziness, confusion, hallucinations, hypertension, tachycardia, chest pain, nausea, and vomiting, which typically have a short duration and require only symptomatic or supportive treatment. Nevertheless, compared with cannabis, complications associated with synthetic cannabinoid use appeared more frequent, and in some cases more severe. Severe clinical complications that have been reported to be associated with synthetic cannabinoid use include convulsions and seizures, status epilepticus, catatonia, delirium, ischemic stroke, intracranial hemorrhage, pulmonary embolism, pneumonia and pulmonary infiltrates respiratory depression, supraventricular and ventricular arrhythmias, myocardial ischemia and infarction, *Takotsubo* cardiomyopathy, liver injury, acute kidney injury, hyperemesis syndrome, and rhabdomyolysis. Furthermore, various psychiatric adverse effects have been reported, including paranoia, psychosis, and ideations of self-harm and suicide (for detailed references, see Luethi and Lichti 2020).

Recent research in Archives of Toxicology focused on cannabinoid toxicokinetics using new analytical methods (Schaefer et al 2020) and on pharmacokinetic–pharmacodynamic (PK–PD) relationships (Lie et al 2021). Such data are of high interest for physicians (Meyer 2016).

A very active field of research is the preclinical research on cannabinoids as illustrated by the following examples:

1. The endocannabinoid system (ECS) seems to be involved also in reproductive process, including ovarian physiology. Female reproductive lifespan is closely related to the number of nongrowing ovarian follicles (the ovarian reserve), which is established during foetal life. Damage of the ovarian reserve may lead to poor reproductive outcome and shortened reproductive lifespan. Castel et al (2020) investigated whether prenatal ECS modulation had an effect on the ovarian reserve at different ages in the rat offspring. Female rats (F0) were exposed to the CB1-/CB2-receptor agonist WIN55212, the CB1R inverse agonist SR141716 or Δ9-THC (5 mg/kg) and were compared to negative control groups. Ovarian reserve was histologically assessed at different postnatal timepoints (PND, F1 individuals). At PND-6, prenatal exposure had no effect on ovarian reserve. In the young adult group (PND-90) exposed during gestation to WIN55212, a CB1R-mediated delayed ovarian reserve decrease was observed, which was reversed by prenatal CB1R blockade by SR141716. After prenatal SR141716 exposure, higher ovarian reserve counts at PND90 were seen. RT-PCR experiments showed that prenatal ECS modulation affected mRNA levels of ECS enzymes and ovarian reserve regulation genes. These findings support the role of the ECS in ovarian reserve regulation during the foetal life of rats.
2. As already outlined above, rhabdomyolysis has been reported in patients abusing synthetic cannabinoids. Tomiyama and Funada (2021) investigated the cytotoxicity of the synthetic cannabinoid CP-55940, a compound that acts equally on both types of cannabinoid receptors (CB1 and CB2), in a human embryonic rhabdomyosarcoma cell line. Exposure of these cells to CP-55940 resulted in concentration-dependent decreases in cell viability. These effects were attenuated by pre-incubation with AM251, a selective CB1 receptor antagonist, but not by pre-incubation with AM630, a selective CB2 receptor antagonist. Following treatment with CP-55940, rhabdomyosarcoma cells exhibited apoptosis, as indicated by the accumulation of annexin-V, activation of caspase-3, and a loss of mitochondrial membrane potential. In addition, CP-55940 treatment of rhabdomyosarcoma cells led to increases in intracellular Ca^2+^ levels. CP-55940-induced cell death was significantly attenuated in the absence of extracellular Ca^2+^, and was partially decreased by pre-incubation with verapamil or diltiazem, compounds that block the L-type Ca^2+^ channel. The results indicate that the cytotoxicity of CP-55940 towards RD cells (skeletal muscle cells) is mediated by the CB1 receptor, but not by the CB2 receptor. Calcium influx through the L-type channel may play a role in the apoptosis induced by these compounds.

In conclusion, 85 years of research illustrate the transformation of Cannabis from an addictive drug to a promising clinical candidate.
